# Description of a new species of *Thelcticopis* Karsch (Araneae, Sparassidae) from Guangxi Province, China

**DOI:** 10.3897/BDJ.9.e67437

**Published:** 2021-06-04

**Authors:** Chaohui Cai, Yejie Lin, Yang Zhong

**Affiliations:** 1 Hubei Key Laboratory of Radiation Chemistry and Functional Materials, School of Nuclear Technology and Chemistry & Biology, Hubei University of Science and Technology, Xianning, Hubei, China Hubei Key Laboratory of Radiation Chemistry and Functional Materials, School of Nuclear Technology and Chemistry & Biology, Hubei University of Science and Technology Xianning, Hubei China; 2 Institute of Zoology, Chinese Academy of Sciences, Beijing, China Institute of Zoology, Chinese Academy of Sciences Beijing China

**Keywords:** biodiversity, huntsman spiders, new species, taxonomy

## Abstract

**Background:**

Due to its special ways of hiding and lifestyle, *Thelcticopis* is a type of spider that is very difficult to collect. In 2018, we collected two huntsman spiders in Guangxi. After comparison with other *Thelcticopis* species, such as anterior median eye larger than other eyes, they were found to belong to the genus of *Thelcticopis*.

**New information:**

Currently, four *Thelcticopis* species are reported from China, *T. severa* (L. Koch, 1875), *T. zhengi* Liu, Li & Jäger, 2010, *T. dahanensis* Zhu & Zhong, 2020 and *T. unciformis* Zhu & Zhong, 2020. They are mainly distributed in the tropical or subtropical areas of China (Fujian, Guangdong, Guangxi, Hainan, Hongkong, Hunan, Taiwan, Yunnan and Zhejiang). In this paper, we diagnose and describe a new species, *Thelcticopis pinmini* sp. nov., which was collected from Guangxi Province, China.

## Introduction

*Thelcticopis* Karsch, 1884 belongs to the subfamliy Sparianthinae Simon, 1897 of the family Sparassidae Bertkau, 1872 (Bertkau 1872, Karsch 1884, Simon 1897). Spiders of *Thelcticopis* are usually medium to large-sized, found on tree bark or inhabiting foliage and leaf litter. They are distributed in Latin and South America, Africa, Asia and Australia (Zhu et al. 2020). Due to their nocturnal lifestyle and quick ways of hiding, matched pairs of species of *Thelcticopis* are hard to collect in the field. To date, currently 50 species have been recorded worldwide, amongst which 37 species are based on a single sex (World Spider Catalog 2021). While examining specimens from Guangxi Province of China, one new species of the genus *Thelcticopis* was identified and is described here.

## Materials and methods

Specimens were examined and measured with a Leica M205C stereomicroscope. Positions of the tegular appendages are given according to clock positions, based on the left palp in ventral view. Male palps were examined after dissection and removal from the spiders’ bodies, the epigynes were examined and illustrated after dissection. Female copulatory organs were cleared in proteinase K at 56°C to dissolve non-chitinous tissues. All photographs were captured with an Olympus C7070 wide zoom digital camera (7.1 megapixels), mounted on an Olympus SZX12 dissecting microscope and assembled using Helicon Focus 7.0 image stacking software. Photographic images were then edited, using Adobe Photoshop CC 2015. Most of the hairs and macrosetae were generally not identified in the palp and epigyne drawings. All specimens are deposited in the Centre for Behavioural Ecology and Evolution, College of Life Sciences, Hubei University, Wuhan, China (CBEE).

Leg measurements in mm are shown as: total length (femur, patella, tibia, metatarsus, tarsus). Number of spines is listed for each segment in the following order: prolateral, dorsal, retrolateral, ventral (in femora and patellae, ventral spines are absent and fourth digit is omitted in the spination formula). Abbreviations: C = conductor; dRTA = dorsal branch of RTA; E = embolus; EA = embolic apophysis; FD = fertilisation duct; TA = tegular apophysis; MS = median septum; mRTA = median branch of RTA; RTA = retrolateral tibial apophysis; S = spermatheca; SP = spermophore; vRTA = ventral branch of RTA.

## Taxon treatments

### Thelcticopis pinmini

Cai & Zhong sp. n.

66F26A9B-CD33-5B65-AE25-58E4AB6444D1

AA1AE18F-9E99-4824-8CAF-8E70361DA535

#### Materials

- **Type status:** Holotype. **Occurrence:** recordedBy: Pinmin Li; individualCount: 1; sex: 1 male; lifeStage: adult; reproductiveCondition: in ethyl alcohol; **Taxon:** scientificName: *Thelcticopis pinmini* sp. nov.; order: Araneae; family: Sparassdiae; genus: Thelcticopis; **Location:** continent: Asian; country: China; countryCode: CHN; stateProvince: Guangxi; county: Fusui; locality: Liuqiao Town; decimalLatitude: -22.31; decimalLongitude: -107.71; **Identification:** identifiedBy: Yang Zhong; **Event:** samplingProtocol: by hand; year: 2018; month: 12; day: 7
- **Type status:** Paratype. **Occurrence:** recordedBy: Pinmin Li; individualCount: 1; sex: 1 female; lifeStage: adult; reproductiveCondition: in ethyl alcohol; **Taxon:** scientificName: *Thelcticopis pinmini* sp. nov.; order: Araneae; family: Sparassdiae; genus: Thelcticopis; **Location:** continent: Asian; country: China; countryCode: CHN; stateProvince: Guangxi; county: Fusui; locality: Liuqiao Town; decimalLatitude: -22.31; decimalLongitude: -107.71; **Identification:** identifiedBy: Yang Zhong; **Event:** samplingProtocol: by hand; year: 2018; month: 12; day: 7

#### Description

**Male.** PL 3.5, PW 3.6, AW 1.6, OL 2.8, OW 2.6. Eyes: AME 0.24, ALE 0.13, PME 0.13, PLE 0.16, AME–AME 0.22, AME–ALE 0.23, PME–PME 0.46, PME–PLE 0.32, AME–PME 0.19, ALE–PLE 0.09, CH AME 0.10, CH ALE 0.22. Spination: Palp: 131, 101, 0002; Fe: I 210, II–IV 311; Pa: I–IV 000; Ti: I–II 2028, III–IV 2026; Mt: I–II 0002, III–IV 2024. Measurements of palp and legs: Palp 5.4 (1.3, 0.7, 1.6, –, 1.8), I 11.7 (3.6, 1.8, 3.1, 2.5, 0.7), II 12.7 (4.1, 2.1, 3.2, 2.6, 0.7), III 10.4 (2.9, 1.4, 2.8, 2.5, 0.8), IV 10.1 (2.9, 1.3, 2.7, 2.4, 0.8). Leg formula: 2-1-3-4. Cheliceral furrow with three anterior and three posterior teeth, without denticles (Fig. 2B). Dorsal prosoma deep reddish-brown, lateral margins dark. Fovea and redial furrows distinctly marked. Labium and gnathocoxae reddish-brown, both with distal parts brighter. Sternum deep reddish-brown, covered by small setae. Legs yellowish-brown with widely dark spots. Dorsal opisthosoma greyish-brown covered by short dark setae. Ventral opisthosoma yellowish-brown covered by densely dark spots (Fig. 4A–B).

**Figure 1.**
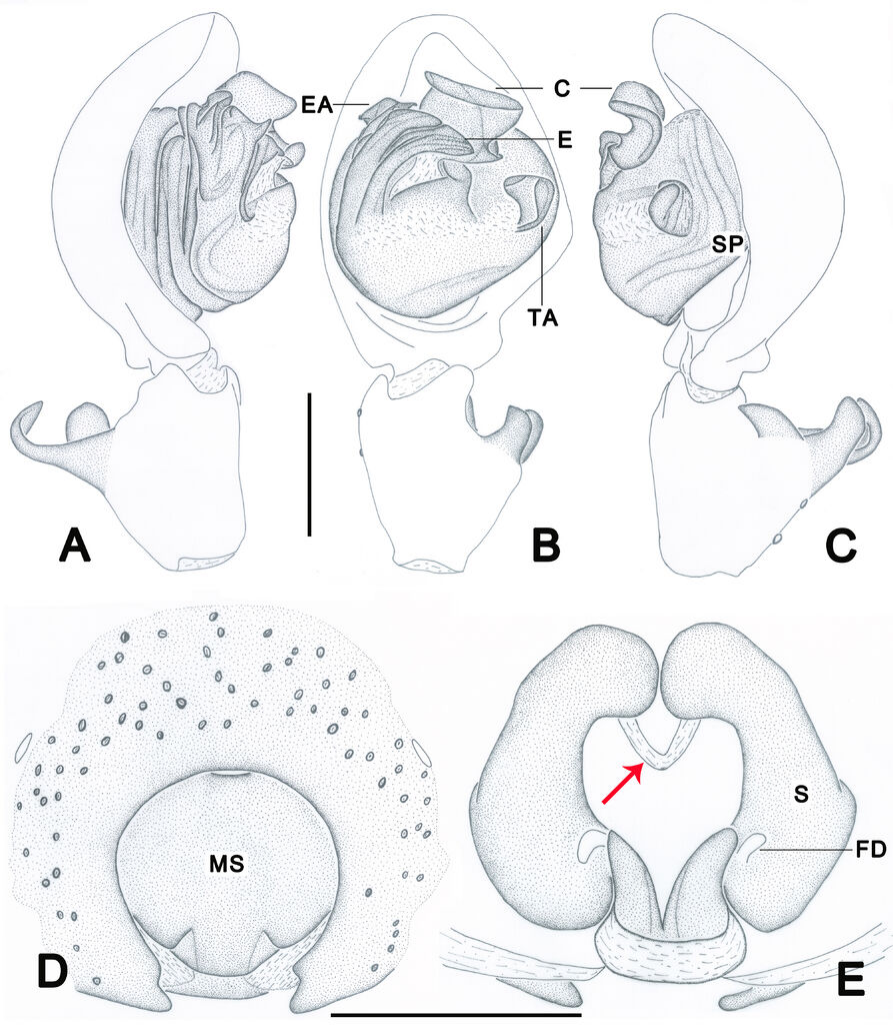
*Thelcticopis pinmini* sp. nov. **A.** Palp, left, ventral view; **B.** Palp, prolateral view; **C.** Palp, retrolateral view; **D.** Epigyne, ventral view; **E.** Vulva, dorsal view. Abbreviations: C—conductor, E—embolus, EA—embolic apophysis, FD—fertilisation duct, MS—median septum, S—spermatheca, SP—spermophore, TA—tegular apophysis. Red arrow: initial part of internal duct system. Scale bars: 0.5 mm.

**Figure 2.**
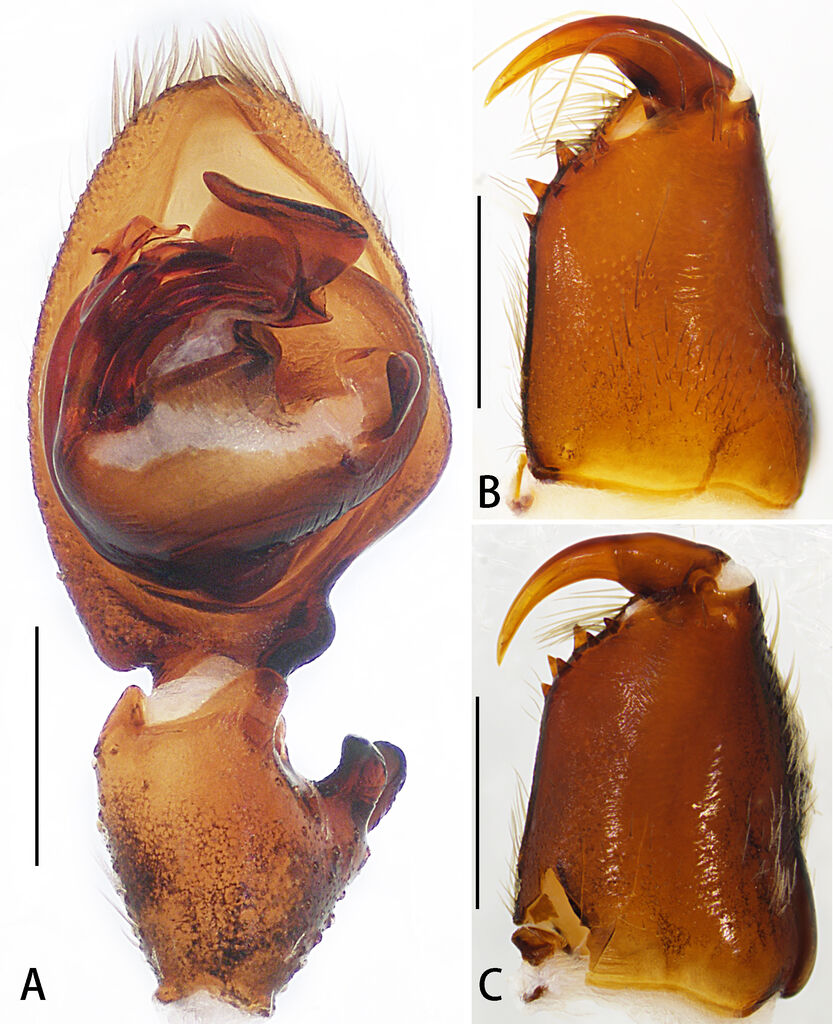
*Thelcticopis pinmini* sp. nov. **A.** Palp, left, ventral view; **B.** Cheliceral dentition, male, ventral view; **C.** Cheliceral dentition, female, ventral view. Scale bars: 0.5 mm.

**Figure 3.**
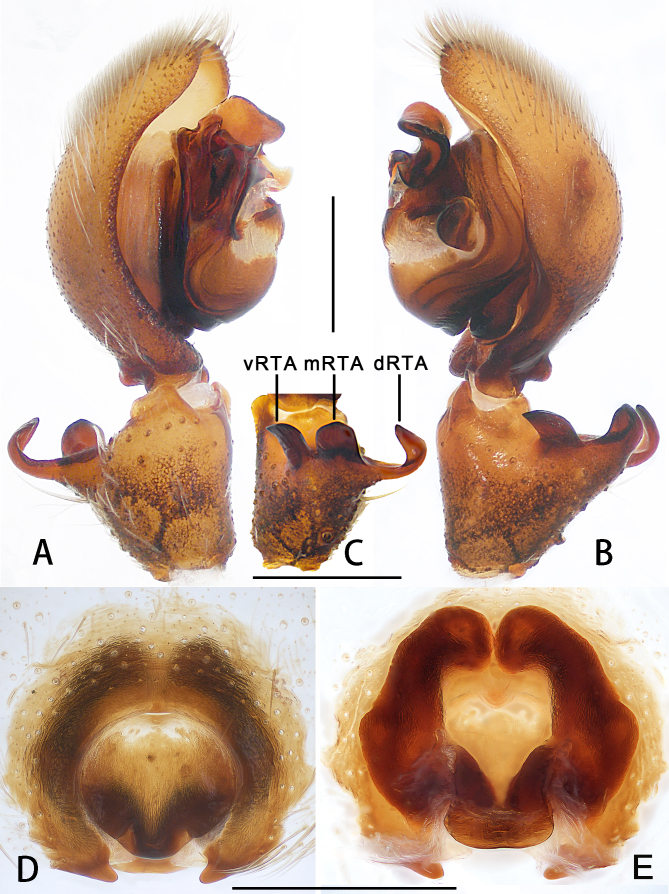
*Thelcticopis pinmini* sp. nov. **A.** Palp, left, prolateral view; **B.** Palp, left, retrolateral view; **C.** Left male palpal tibia, dorsal view; **D.** Epigyne, ventral view; **E.** Vulva, dorsal view. Abbreviations: dRTA—dorsal branch of RTA, mRTA—median branch of RTA, vRTA—ventral branch of RTA. Scale bars: 0.5 mm.

Palp as in diagnosis. Cymbium approximately two times longer than tibia in ventral view. Embolus arising from tegulum at 9-o’clock-position, with embolic tip distinctly wider than embolic apophysis. Conductor arising from tegulum in 12-o’clock-position. Tegular apophysis spoon-shaped, best seen in ventral view. Spermophore distinctly curved in retrolateral view. RTA arising medially from tibia, with three apices best seen in dorsal view. dRTA distinctly curved, U-shaped in prolateral view (Fig. 1A–C, Fig. 2A and Fig. 3A–C).

**Female.** PL 3.6, PW 3.5, AW 1.6, OL 4.7, OW 3.2. Eyes: AME 0.22, ALE 0.18, PME 0.14, PLE 0.19, AME–AME 0.20, AME–ALE 0.23, PME–PME 0.39, PME–PLE 0.36, AME–PME 0.22, ALE–PLE 0.08, CH AME 0.18, CH ALE 0.22. Spination: Palp: 131, 101, 2221, 1011; Fe: I–II 220 III–IV 120; Pa: I–IV 000; Ti: I–II 0008, III 0006, IV 0004; Mt: I–III 0002, IV 1014. Measurements of palp and legs: Palp 3.2 (0.9, 0.6, 0.7, –, 1.0), I 8.9 (2.9, 1.5, 2.4, 1.5, 0.6), II 9.6 (3.2, 1.7, 2.6, 1.5, 0.6), III 8.4 (2.9, 1.4, 2.0, 1.5, 0.6), IV 8.7 (2.9, 1.3, 2.4, 1.4, 0.7). Leg formula: 2-1-4-3. Cheliceral furrow with three anterior and four posterior teeth, without denticles (Fig. 2C).

Copulatory organs as in diagnosis. Epigynal field wider than long with one slit sensillum on each side of the epigynal field. Median septum medially about 1/2 of epigynal's width, with two humps on the posterior part. Posterior margin with two hooks, each pointing medially. Both sides of internal duct sustem separated. Fertilisation ducts arising posterior-laterally (Fig. 1D–E and Fig. 3D–E).

**Figure 4.**
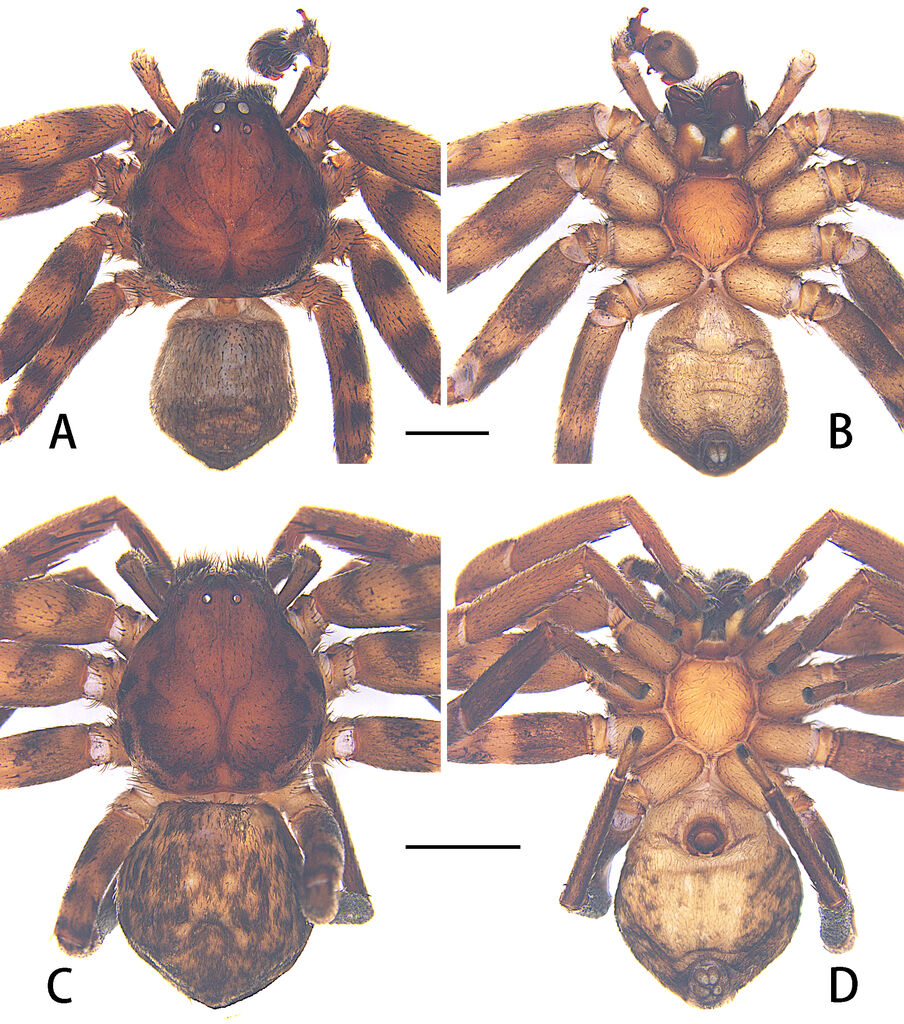
Habitus of *Thelcticopis pinmini* sp. nov. **A.** Male, ventral view; **B.** Male, dorsal view; **C.** Female, ventral view; **D.** Female, dorsal view. Scale bars: 2 mm.

Colouration in ethanol: as in male, but dorsal opisthosoma yellowish-brown with wide dark spots (Fig. 4C–D).

#### Diagnosis

Male of this species can be distinguished from the remaining species of the genus by its palps with embolic apophysis and embolus distally with an obvious crack (Fig. 1A-C, Fig. 2A and Fig. 3A-B). Female resembles *Thelcticopis folia* Jäger & Praxaysombath, 2009 (see Jäger and Praxaysombath 2009: 48, figs. 106–113) in having a roundish median plate (Fig. 1D and Fig. 3D) and initial part of internal duct system narrow, leading to a chamber (Fig. 1E and Fig. 3E), but can be distinguished from the latter by the following characters: 1, median septum rounded in anterior part and two humps on the posterior part (not in *T. folia*); 2, anterior part of internal duct system touching each other and distinctly narrower than posterior part (separated, the median part narrower than the other parts in *T. folia*).

#### Etymology

The specific name is dedicated to Mr Pinmin Li for his kind instructions on our collection of huntsman spiders; name in the genitive case.

#### Distribution

**Figure 5.**
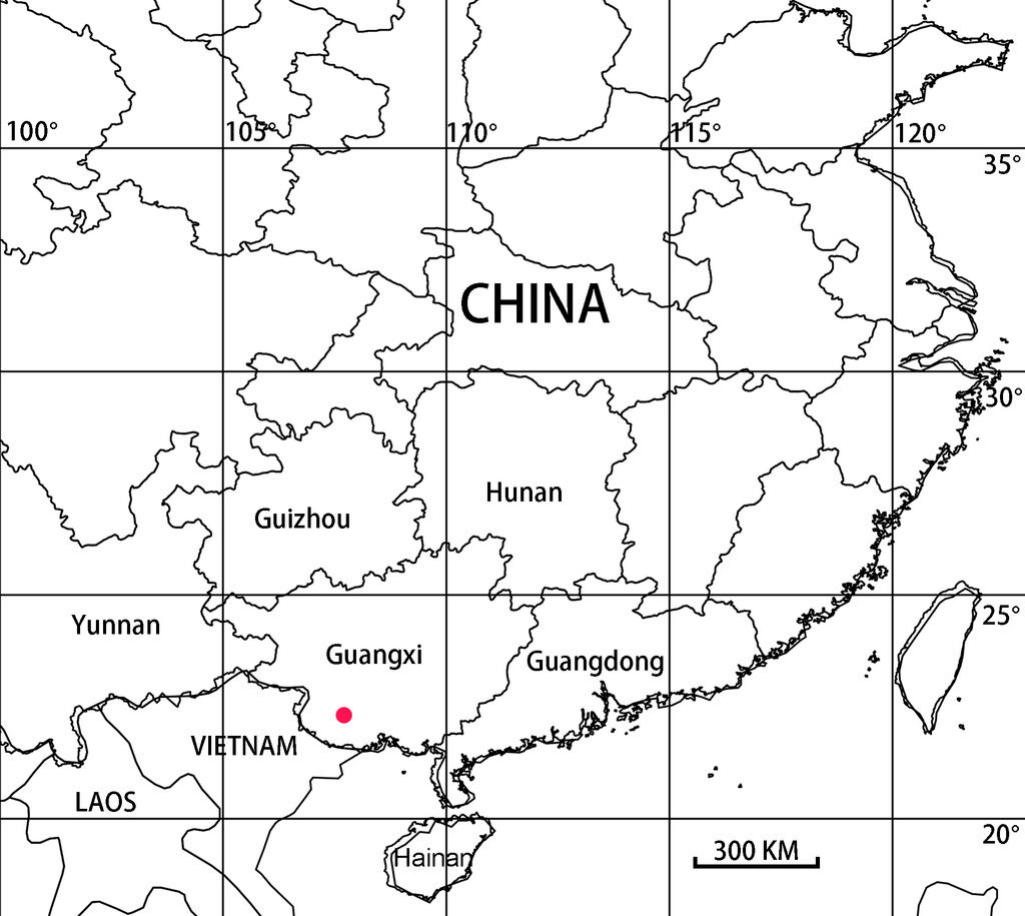
Distribution of *Thelcticopis pinmini* sp. nov.

Known only from the type locality (Fig. 5).

## Supplementary Material

XML Treatment for Thelcticopis pinmini
